# Evolutionary ecology of the visual opsin gene sequence and its expression in turbot (*Scophthalmus maximus*)

**DOI:** 10.1186/s12862-021-01837-2

**Published:** 2021-06-07

**Authors:** Yunong Wang, Li Zhou, Lele Wu, Changbin Song, Xiaona Ma, Shihong Xu, Tengfei Du, Xian Li, Jun Li

**Affiliations:** 1 College of Fisheries, Ocean University of China, Qingdao, 266003 People’s Republic of China; 2 CAS Key Laboratory of Experimental Marine Biology, Institute of Oceanology, Chinese Academy of Sciences, Qingdao, 266071 People’s Republic of China; 3 Laboratory for Marine Biology and Biotechnology, Qingdao National Laboratory for Marine Science and Technology, Qingdao, 266071 People’s Republic of China; 4 Center for Ocean Mega-Science, Chinese Academy of Sciences, Qingdao, 266071 PR China; 5 Institute of Semiconductors, Chinese Academy of Science, Beijing, 100083 People’s Republic of China

**Keywords:** Turbot, Benthic life, Adaption, Opsin, Heterochronic shift

## Abstract

**Background:**

As flatfish, turbot undergo metamorphosis as part of their life cycle. In the larval stage, turbot live at the ocean surface, but after metamorphosis they move to deeper water and turn to benthic life. Thus, the light environment differs greatly between life stages. The visual system plays a great role in organic evolution, but reports of the relationship between the visual system and benthic life are rare. In this study, we reported the molecular and evolutionary analysis of opsin genes in turbot, and the heterochronic shifts in opsin expression during development.

**Results:**

Our gene synteny analysis showed that subtype *RH2C* was not on the same gene cluster as the other four green-sensitive opsin genes (*RH2*) in turbot. It was translocated to chromosome 8 from chromosome 6. Based on branch-site test and spectral tuning sites analyses, E122Q and M207L substitutions in *RH2C*, which were found to be under positive selection, are closely related to the blue shift of optimum light sensitivities. And real-time PCR results indicated the dominant opsin gene shifted from red-sensitive (*LWS*) to *RH2B1* during turbot development, which may lead to spectral sensitivity shifts to shorter wavelengths.

**Conclusions:**

This is the first report that *RH2C* may be an important subtype of green opsin gene that was retained by turbot and possibly other flatfish species during evolution. Moreover, E122Q and M207L substitutions in *RH2C* may contribute to the survival of turbot in the bluish colored ocean. And heterochronic shifts in opsin expression may be an important strategy for turbot to adapt to benthic life.

**Supplementary Information:**

The online version contains supplementary material available at 10.1186/s12862-021-01837-2.

## Background

To survive, all organisms must react to changes in the physical environment, such as temperature, circadian rhythm of light, and humidity [1]. Thus, to frame the evolutionary perspective about the molecular basis of organismal adaptation and biology, sensory systems are generally selected as ideal models [2]. Vision is closely related to behaviors such as foraging, mating, parental care, and avoiding predation. As it allows for almost instantaneous transmission of information, vision likely plays a great role in diversification [3]. Vision formation involves retinal reception, integration, and higher-order brain processing [4, 5]. Retinal reception is mediated by visual pigments, which consist of one opsin protein, a group of G protein-coupled receptors, and one chromophore (11-*cis*-retinal, A1, or 11-*cis*-3, 4-dehydroretinal, A2) [3].

Due to water absorption and scattering of light, the photic environment of the aquatic system in which fish live changes rapidly with depth, especially a shift to predominantly blue wavelengths in the ocean at about 60–75 depth [1, 2]. Teleost code five classes of visual opsin genes: (1) *RH1* (rhodopsin, spectral peak absorbances around 500 nm) for dim light; (2) *RH2* (rhodopsin-like opsin, 470–510 nm) for green; (3) *SWS1* (short wavelength-sensitive type 1, 360–430 nm) for ultraviolet; (4) *SWS2* (SWS1-like opsin, 440–460 nm) for blue; and (5) *LWS* (long wavelength-sensitive, 510–560 nm) for red. With the exception of *RH1*, which is expressed in rod photoreceptors, the other four classes are expressed in cone photoreceptors [3]. The cone opsin genes derived from two rounds of whole genome duplication [6]. It is believed that after several additional duplication events, diverse opsin repertoires were maintained among different species [7–12]. *RH2* and *LWS* duplication events were most prevalent in ray-finned fish, and tandem duplication seems to have produced more duplicates [13]. Due to substitution in key sites, the multiple opsin subtypes from duplication generally have different spectral peak absorbances (λ_max_), which helps enrich the visual system [2, 3, 7, 10].

In addition to the adaptive evolution of gene sequences, heterochronic shifts in visual opsin expression are an important mechanism of spectral tuning [14, 15]. For example, single cones in rainbow trout (*Oncorhynchus mykiss*) switch opsin expression from *SWS1* to *SWS2* during the juvenile period [16], and in winter flounder (*Pleuronectes americanus*), only *RH2* is expressed in the pre-metamorphic retina, whereas *RH1*, *SWS2*, and *LWS* are also expressed in the post-metamorphic retina [17]. Besides ontogenetic changes, plasticity of visual opsin expression in response to different photic environments is an important strategy that allows rapid adaptation to environmental changes. Plastic opsin expression was reported to have a profound effect on Nile tilapia (*Oreochromis niloticus*) and guppy (*Poecilia reticulate*) during development. Specifically, juveniles and adults *O. niloticus* differ in spectral reflectance after the two environmental light treatments, demonstrating that environmental light plays a great role in signal production throughout ontogeny. And developmental plasticity in vision may help *P. reticulate* overcome increased turbidity [18, 19]. It was also reported for the adult stage of fish such as red shiner (*Cyprinella lutrensis*) and bluefin killifish (*Lucania goodei*) [20, 21]. Additionally, a recent study showed that opsin coexpression might be a novel mechanism for modulating color vision [22]. However, it is unclear whether the direction and extent of opsin expression plasticity is limited by ontogeny [23].

The turbot (*Scophthalmus maximus*) is an important aquaculture species with great commercial value. As a flatfish, metamorphosis is a critical part of its life cycle [24, 25]. During early life stage, *S. maximus* larvae live at the ocean surface and undergo metamorphosis characterized by asymmetrical body transformation coupled with eye migration. After metamorphosis, turbot move to deep water and enter a benthic phase [26, 27]. This change in habitat results in a great shift in environmental conditions, and its visual system may change accordingly. For example, the photoreceptors in the retina are mainly composed of cone cells before metamorphosis, while during metamorphosis the rod cells increase and become the main component of photoreceptors in pre-metamorphic phase [28, 29]. However, visual characteristics and the opsin expression pattern of turbot and their relationship to benthic life remain unknown. In the present study, we investigated the selective pressure acting on turbot and eight other teleost species and conducted spectral tuning site and synteny analyses to evaluate the adaptive evolution of turbot visual opsin genes. We also investigated the heterochronic shifts during development of turbot. Results of this study enrich the understanding of sensory adaption in demersal fish.

## Results

### Phylogeny and syntenic analysis of turbot visual opsin

**Fig. 1:**
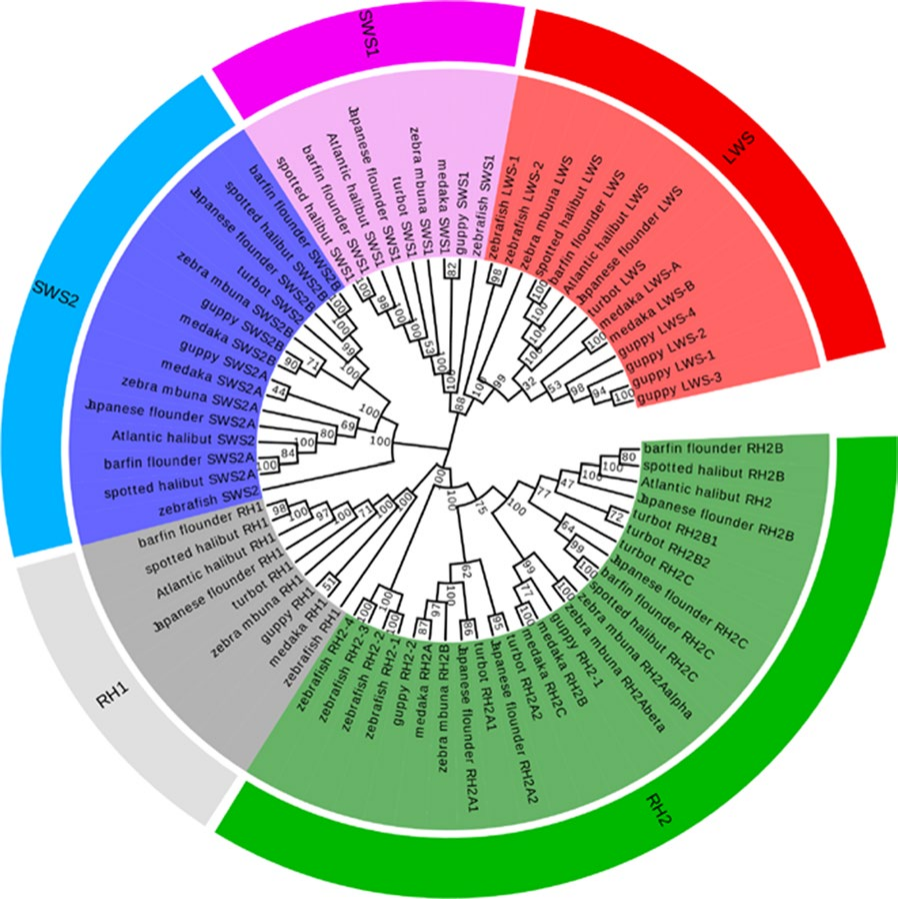
Phylogenetic relationships of the turbot opsin genes and other teleost opsin genes based on the maximum-likelihood method. The bootstrap test (1000 replicates) scores are shown on the nodes

**Fig. 2:**
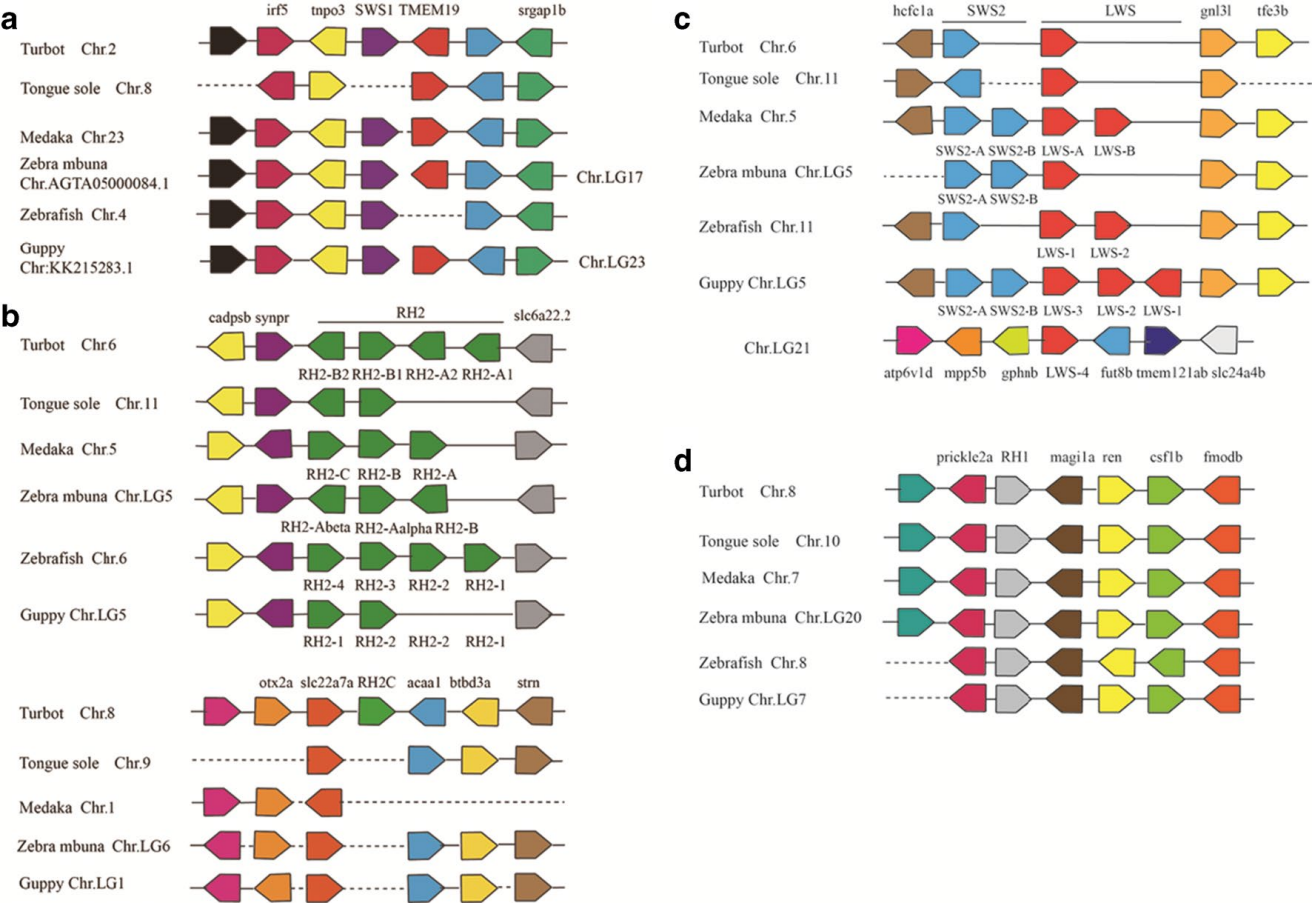
Synteny analyses of visual opsin genes between turbot and five other teleost species (tongue sole, medaka, zebra mbuna, zebrafish, and guppy). **a** *SWS1*, **b** *RH2*, **c** *LWS* and *SWS2*, **d** *RH1*. Different gene families are represented by colored pentagons, and the direction of the pentagon indicates gene orientation. The dashed lines indicate that two adjacent genes in that species are not directly linked

We obtained an unrooted visual opsin phylogenetic tree of turbot and eight other species constructed using the maximum-likelihood method (Fig. 1). The tree confirmed the identities of turbot opsin genes: *RH1*, *SWS1*, *SWS2*, *RH2A1*, *RH2A2*, *RH2B1*, *RH2B2*, *RH2C*, and *LWS*. Unlike other single-copy opsin genes, turbot have five *RH2* paralogs. Figure 2 shows the results of comparative synteny analyses among six selected teleost species. In general, opsin gene positions were conserved among teleosts. For example, the *RH1* gene was typically positioned between the gene loci of *prickle2a* and *ren*. In addition to *RH2C*, four other *RH2* genes of turbot were located in tandem on chromosome 6. By comparing the syntenic region containing *RH2C*, we found that although the genomic regions both downstream and upstream were otherwise maintained, *RH2C* was missing in the other five species (Fig. 2b). When we extended the analysis to all fish genomes that could be analyzed, the *RH2C* locus was still not found. Similarly, *LWS-1*, *LWS-2*, and *LWS-3* of guppy formed a tandem gene cluster, while *LWS-4* was located on chromosome LG21 (Fig. 2c). Additionally, *SWS1* was not present in the tongue sole genome (Fig. 2a).

### Natural selection and spectral tuning sites analysis

To evaluate the selective constraints acting at the branch level in teleosts, branch-specific selection analysis was performed. In all cases of opsin genes, the free-ratio model provided a better fit, indicating the heterogeneity of ω values (nonsynonymous/synonymous rate ratio, ω = *dN*/*dS*) among branches (Table 1). For the branch-site models, the test was conducted on a particular branch of the tree. The LRT comparisons between model A and the null model revealed several sites under positive selection on nine branches of the tree (red labeled branches in Fig. S1). These sites under episodes of positive selection are listed in Table 2. Among the lineages selected in the phylogenetic tree, only LWS lacked a positive selection site. Furthermore, positive results were found for all branches of zebrafish except LWS. Among all selected branches, the SWS2 branch of zebrafish had the most sites.

**Table 1:** Statistics of branch-specific model analysis of turbot and other teleost species

Gene	lnL (1ω model)	lnL (free ω model)	Likelihood ratio test		*P* Values
*LWS*	− 4964.165032	− 4922.267355		83.795354	< 0.001
*SWS1*	− 4253.724995	− 4240.774669		25.900652	0.0391
*SWS2*	− 8001.473925	− 7970.454002		62.039846	< 0.001
*RH2*	− 10,370.70927	− 10,296.73427		147.949996	< 0.001
*RH1*	− 3734.012013	− 3705.674753		56.67452	< 0.001

The likelihood ratio test is to determine whether the free ω model fits the data significantly better than the one ω model. The ω for each branch are not shown. *lnL* ln likelihood

**Table 2:** Parameter estimates of branch-site models and predicted positively selected sites

Opsin	Clade	lnL		LRT	*P*	Positively selected sites
		Null	modelA			
RH2	a	− 10,169.10	− 10,169.10	0	1	–
	b	− 10,166.54	− 10,164.23	4.62	0.0315	19 (0.881) 31 (0.628) 95 (0.945) 109 (0.882) 122 (0.511) 169 (0.501) 207 (0.966) 266 (0.604) 273 (0.949) 320 (0.578)
	c	− 10,169.10	− 10,169.10	0	1	–
	d	− 10,167.62	− 10,165.03	5.16	0.0230	105 (0.612) 217 (0.993)
	e	− 10,168.60	− 10,168.58	0.03	0.8636	–
	f	− 10,166.92	− 10,162.26	9.32	0.0022	49 (0.944) 95 (0.733) 270 (0.982) 307 (0.907)
	g	− 10,148.85	− 10,136.84	24.01	< 0.001	14 (0.875) 33 (0.514) 50 (0.946) 65 (0.770) 84 (0.948) 151 (0.523) 195 (0.951) 197 (0.845) 198 (0.600) 217 (0.945) 239 (0.528) 254 (0.952) 286 (0.502) 290 (0.594) 297 (0.581) 321 (0.757) 322 (0.966) 323 (0.990) 325 (0.979) 328 (0.649) 329(0.770) 330 (0.772)
RH1	a	− 3629.453904	− 3625.696675	7.51	0.0061	35 (0.769) 126 (0.674) 196 (0.858) 210 (0.879) 235 (0.911) 236 (0.959) 281 (0.773)
	b	− 3632.72	− 3632.72	0	1	–
	c	− 3632.72	− 3632.72	0	1	–
	d	− 3630.82	− 3630.82	4E-06	1	–
SWS2	a	− 7840.16	− 7831.84	16.63	< 0.001	2 (0.572) 5 (0.605) 10 (0.815) 14 (0.801) 15 (0.575) 21 (0.841) 22 (0.579) 25 (0.507) 27 (0.805) 42 (0.552) 44 (0.889) 45 (0.598) 51 (0.605) 55 (0.623) 87 (0.570) 88 (0.617) 91 (0.567) 95 (0.613) 99 (0.510) 123 (0.556) 125 (0.539) 156 (0.601)162 (0.619) 166 (0.598) 244 (0.600) 257 (0.600) 269 (0.670) 279 (0.604) 283 (0.853) 301 (0.685) 315 (0.708) 328 (0.858)329 (0.818) 332 (0.577) 335 (0.596) 339 (0.533) 341 (0.545) 346 (0.659) 349 (0.687)
	b	− 7851.02	− 7848.07	5.88	0.0152	38 (0.560) 99 (0.904) 276 (0.687) 299 (0.983)
	c	− 7852.17	− 7850.19	3.97	0.0463	18 (0.894)
	d	− 7852.21	− 7852.21	0	1	–
	e	− 7852.13	− 7852.10	0.06	0.8135	–
	f	− 7852.21	− 7851.96	0.50	0.4798	–
SWS1	a	− 4194.06	− 4194.06	0	1	–
	b	− 4194.59	− 4193.82	1.54	0.2146	–
	c	− 4189.36	− 4185.92	6.87	0.0087	8 (0.895) 9 (0.860) 10 (0.788) 11 (0.765) 16 (0.704) 27 (0.895) 56 (0.678) 70 (0.888) 125 (0.746) 145 (0.751) 185 (0.881) 194 (0.766) 263 (0.792) 342 (0.512)
LWS	a	− 4828.06	− 4828.06	0	1	–
	b	− 4828.06	− 4828.06	0	1	–
	c	− 4828.06	− 4828.06	0	1	–
	d	− 4828.06	− 4828.06	0	1	–

*lnL* ln likelihood, *LRT* likelihood ratio test, – no positively selected sites

Based on amino acid multiple alignments, we surveyed the main tuning sites involved in spectral sensitivity. Tables 3, 4 show the results for RH2 and RH1, respectively, and the others are shown in Additional file 1: Tables S2–4. Asparagine (N) was present at site 83 in five flatfish species, while the other teleost genomes had aspartic acid (D) in RH1; the other three sites have yet to be defined. With the exception of Atlantic halibut, the other four flounders all had changes of glutamic acid (E) to glutamine (Q) at site 122 and methionine (M) to leucine (L) at site 207 in RH2C. For SWS1, sites 46 and 114 of turbot contained phenylalanine (F) and alanine (A), respectively, but serine (S) was present in those locations for the other four flatfish species. Moreover, in the b branch of SWS2 in the tree (Additional file 1: Fig. S1), all four species had the same amino acid substitution of valine (V) for threonine (T) at site 99. We also found a turbot-specific amino acid site (122S) in SWS2.

**Table 3:** Comparison of representative spectral tuning sites among teleost RH2 opsins

Tuning site	97	122	207	292
Spotted halibut				
RH2B	S	E	M	A
RH2C	T	*Q*	*L*	A
Barfin flounder				
RH2B	S	E	M	A
RH2C	T	*Q*	*L*	A
Atlantic Halibut				
RH2	S	E	M	A
Japanese flounder				
RH2A1	S	Q	M	A
RH2A2	T	Q	M	A
RH2B	S	E	M	A
RH2C	S	*Q*	*L*	A
Turbot				
RH2A1	S	Q	L	A
RH2A2	T	Q	M	A
RH2B1	S	E	M	A
RH2B2	S	E	M	A
RH2C	S	*Q*	*L*	A
Cichlids				
RH2Aα	S	E	M	A
RH2Aβ	S	E	M	A
RH2B	S	Q	M	A
Medaka				
RH2A	S	Q	M	S
RH2B	S	E	M	A
RH2C	S	Q	M	A
Guppy				
RH2-1	S	E	M	A
RH2-2	S	Q	L	A
Zebrafish				
RH2-1	C	Q	M	A
RH2-2	C	Q	M	A
RH2-3	T	Q	M	A
RH2-4	T	E	M	A

Marked in Italics are two positively selected sites of RH2C

**Table 4:** Comparison of representative spectral tuning sites among teleost RH1 opsins

Tuning site	83	122	261	292
Spotted halibut	N	E	F	A
Barfin flounder	N	E	F	A
Atlantic Halibut	N	E	F	A
Japanese flounder	N	E	F	A
Turbot	N	E	F	A
Medaka	D	E	F	A
Guppy	D	E	F	A
Cichlids	D	E	F	A
Zebrafish	D	E	F	A

### Divergence time of turbot *RH2* genes

**Fig. 3:**
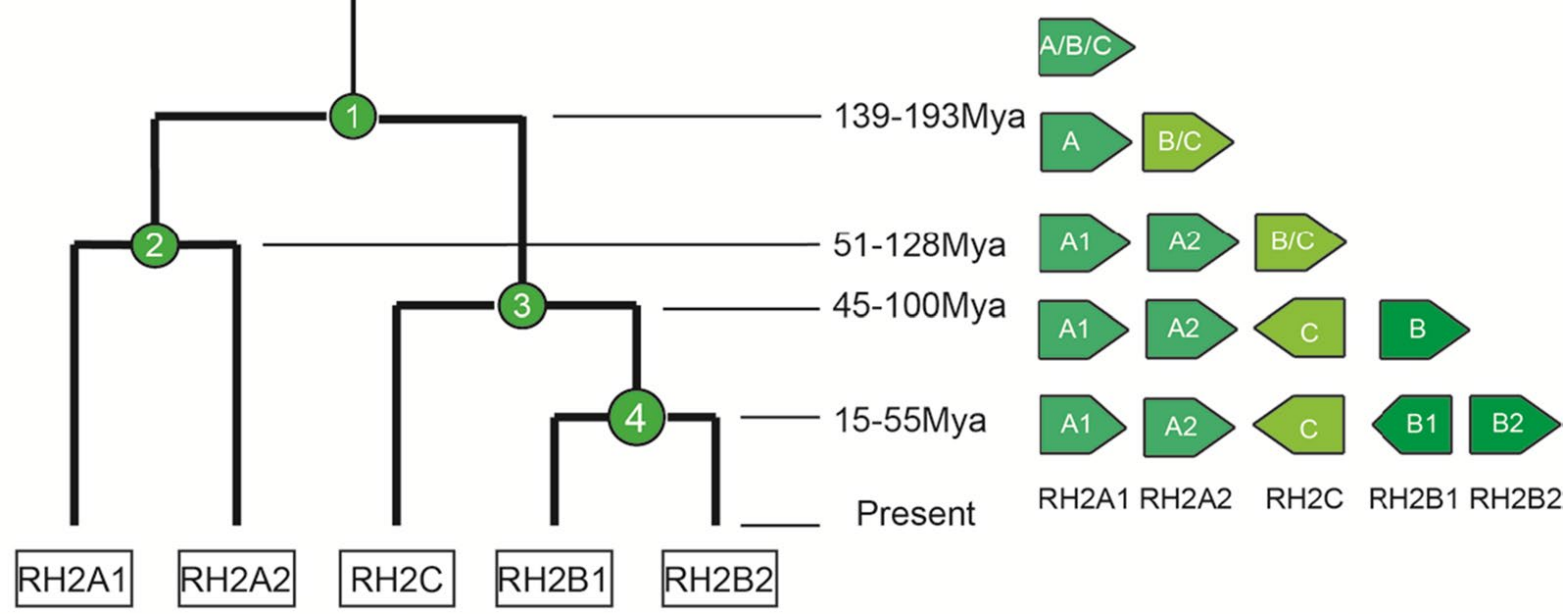
An inferred evolutionary pathway of turbot *RH2* genes. The number on each node of the dendrogram (left) represents a duplication event, whereas the right side shows the changes in *RH2* gene orientations during evolution

The time that the turbot *RH2* genes diverged was estimated by MCMCTree using the soft fossil constraints method, and then we obtained an evolutionary pathway (Fig. 3). We speculated that five *RH2* genes were the product of several duplication events. The proposed first divergence time was 166 (139–193) million years ago (Mya) in the Jurassic, which formed *RH2A* and *RH2B*/*RH2C*. The third divergence (about 71 Mya) created *RH2B* and *RH2C*. The divergence times of *RH2A1*/*RH2A2* and *RH2B1*/*RH2B2* were estimated to be 87 (51–128) and 34 (15–55) Mya, respectively. The divergence of *RH2B1*/*RH2B2* was the latest one, and it occurred in the Tertiary.

### Ontogenetic shift of opsin gene expression in turbot

**Fig. 4:**
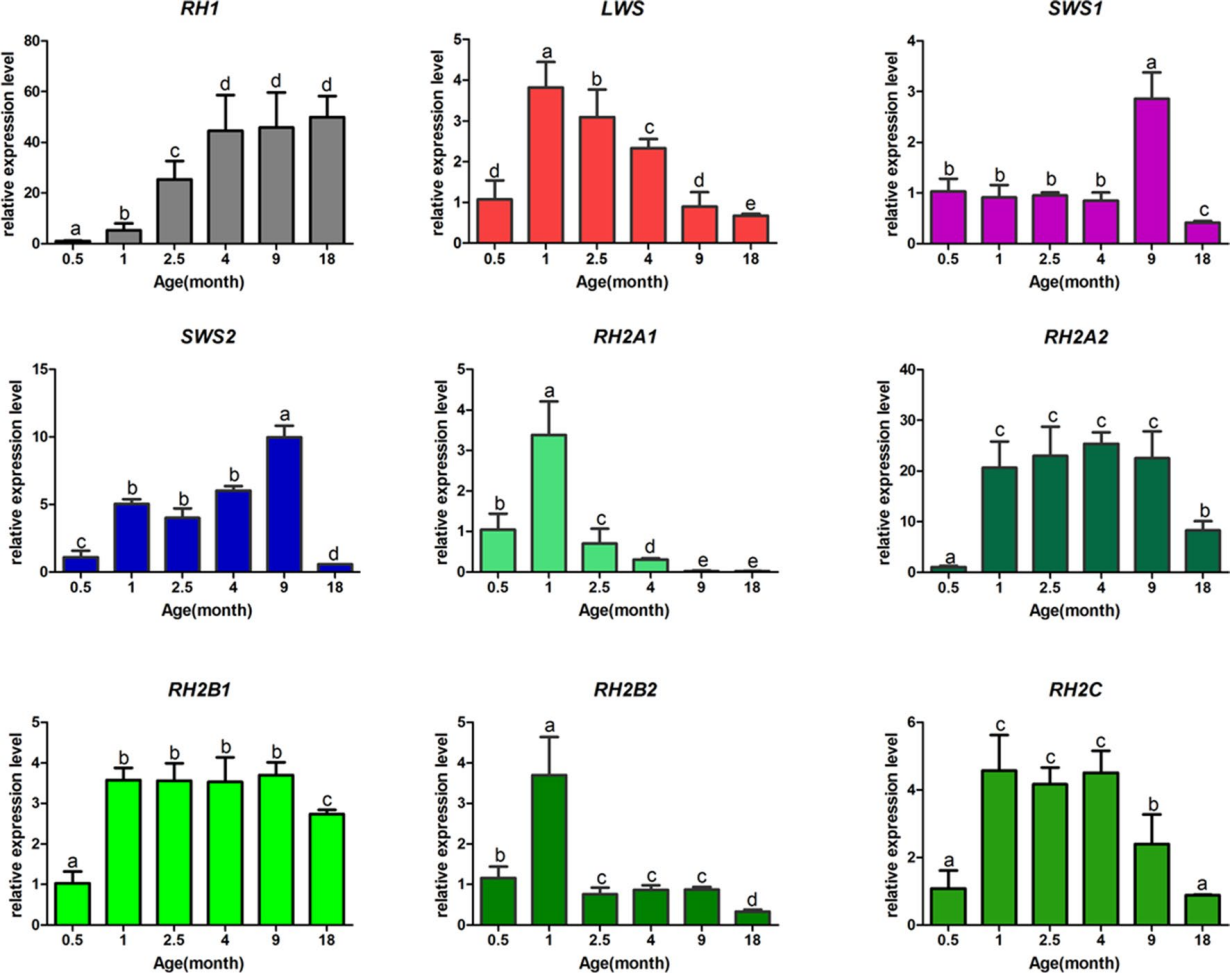
Visual opsin expression of turbot at different stages. Gene expression was measured by quantitative Real-Time PCR (qPCR) with the TB Green Premix Ex Taq assay, and mRNA expression levels of each gene were averaged over several individuals: 0.5 month [15 days post hatching (dph), n > 30], 1 month (30 dph, n > 30), 2.5, 4, 9, and 18 months (n = 3). Different letters represent statistically significant differences between stages (P < 0.05)

In these experiments, expression levels of visual opsin genes in turbot at different developmental stages were determined by qPCR. Eight of the visual opsin genes were expressed at a low level at 0.5 and 18 months of age, but *RH1* was highly expressed at 18 months of age. From 1 to 9 months of age, *RH1*, *SWS2*, and *SWS1* expression significantly increased and *LWS*, *RH2A1*, *RH2B2*, and *RH2C* expression significantly decreased as turbot grew. No significant change was detected in *RH2A2* and *RH2B1* gene expression throughout ontogeny from 1 to 9 months age (Fig. 4). From 4 to 18 months of age, *RH1* expression maintained consistently at a high-level. The expression levels of *SWS1* and *SWS2* at 9 months of age could be twice as much as that at 1 month of age. However, the expression levels of them at 18 months of age are both very low. In the case of *LWS*, its expression level gradually decreased during the development of 4–18 months of age. Among the *RH2* genes, *RH2A1*/*RH2B2* and *RH2A2*/*RH2B1* exhibited the same expression pattern throughout ontogeny, and the pattern for *RH2C* was close to that of *RH2A2* and *RH2B1*. When we analyzed the proportional opsin gene expression of each cone opsin gene, we found that *LWS*, *RH2B1*, and *RH2C* were the three genes present in the highest proportions. The maximum LWS expression level was 53.9% at 1 month of age, while that of *RH2B1* was 63.3% at 9 months of age. Thus, the dominant opsin shifted from *LWS* to *RH2B1* during turbot development (Table 5).

**Table 5:** Proportional expression of cone opsin genes of turbot at different stages

Age (month)	*LWS*	*RH2B1*	*RH2C*	*Other*
0.5	47.98^a^	28.13^a^	16.12^a^	7.77^a^
1	53.85^a^	26.14^a^	13.29^a^	6.71^ab^
2.5	47.43^a^	34.13^a^	14.51^a^	3.93^b^
4	24.71^b^	53.39^b^	17.35^a^	4.55^b^
9	14.64^c^	63.27^b^	18.74^a^	3.35^ab^
18	29.22^b^	57.19^b^	11.22^b^	2.37^b^

Different letters represent statistically significant differences between stages (*P* < 0.05)

## Discussion

### Molecular evolution of RH2 and RH1

Through phylogenetic analysis, we characterized the visual opsin genetic component of turbot. Five green opsin genes are present in the turbot genome, and this is the same number as Pacific bluefin tuna (*Thunnus orientalis*) [10, 30]. Our gene synteny analysis showed that *RH2C* was not in the same gene cluster as the other four *RH2* genes. In addition, the *RH2C* locus was absent in other teleost species analyzed in this study. Unfortunately, our gene synteny analysis failed to cover all species of fish, including some flatfish such as barfin flounder, spotted halibut, and Japanese flounder, so we do not know yet whether their *RH2C*s are arranged in tandem with other *RH2* genes on the chromosome. Additionally, despite the close genetic relationship of tongue sole, it lacks the *RH2C* locus. We predicted that *RH2C* might have originated from flatfish-specific duplication, after which it was translocated to chromosome 8 from chromosome 6 in turbot. It is generally accepted that gene duplication may generate redundant genes, which is usually followed by degenerative mutations in one member of the pair and even gene loss [13, 31, 32]. Subsequently, some flatfish lineages lost *RH2C*, whereas other species such as turbot retained it.

We detected 10 positive selection sites of RH2C, and sites 122 and 207 are related to spectral sensitivity. In barfin flounder, the λ_max_ of RH2C is blue shifted by approximately 16 nm compared to RH2B [33]. Additionally, mutagenesis experiments in coelacanth indicated that both E122Q and M207L cause blue shifts of spectral peak absorbances [3, 34, 35]. Thus, we speculated that the λ_max_ of RH2A1, RH2A2, and RH2C are blue shifted in turbot. Moreover, in turbot, multiple copies of RH2 encode green opsins with different λ_max_, as is found in zebrafish and medaka [7, 8]. Different spectral peak absorbances are beneficial because they allow the fish to discriminate a wider spectrum of light. It may enhance color vision and contribute to prey detection in the bluish ocean [10]. Based on the loss of *RH2A* function in the genus *Verasper* [11] and our results showing low expression levels of *RH2B2* and pairs of *RH2A*, we deduce that *RH2B1* and *RH2C* are the higher expressed *RH2* genes in turbot. Furthermore, the retention of *RH2C* is an adaptation of turbot to the spectral environment in the deep sea due to its shortwave-shift of λ_max_. Additionally, some studies have shown that opsin genes are tied to nuptial and body coloration [36, 37], but further work is required to confirm this function. Regarding the *RH1* genes, all flatfish species studied herein have the substitution of D83N, which has been demonstrated to cause blue shift in cattle and chameleons [38]. However, this site was not positively selected. Therefore, whether this replacement is beneficial for benthic adaptation needs further study. For the other three sites, no differences were found in nine fish species [39–41].

### Heterochronic changes in opsin gene expression

Heterochronic changes in a variety of traits have been reported, including opsin gene expression [15, 42–45]. A study of cichlids revealed that subfunctionalization of heterochronic changes in expression patterns is critical for preservation of opsin genes [46]. Furthermore, altering opsin expression patterns during ontogeny is an important mechanism for modulating color vision [14]. In order to adapt to different life cycle stages, organisms show three patterns of heterochronic changes in opsin gene expression. For instance, in cichlid fishes, Nile tilapia showed a normal pattern of opsin gene expression that changes dynamically from a larval gene set to a final adult set. In contrast, cichlids from Lake Victoria had only an adult gene set with little change through time (direct developing pattern), and rock dwellers from Lake Malawi had a reduced rate of change (neotenic pattern) [15]. In the current study, we found a normal pattern of visual opsin expression in turbot. Turbot undergo metamorphosis during growth and development, which is accompanied by changes in the spectral environment: the bright daylight and long wavelength spectrum environment during the pelagic phase and the dim light and short to medium wavelength spectrum environment in the benthic phase. Transformation of light environments in turn might lead to shifts in opsin gene expression and spectral sensitivity [47]. Our results showed that turbot undergo heterochronic shifts in opsin gene expression, which may alter spectral sensitivity and contribute to scotopic vision. Specifically, the increased expression level of *RH1* is beneficial for vision in diminishing luminance when the fish moves to deeper waters. As for cone opsin genes, the highest proportional expression level of *LWS* at the early developmental stages was gradually replaced by *RH2B1* at the later developmental stages during ontogeny. This variation means that the visual sensitivity shifts from red to green during adaptation to the deep sea environment. Downregulation of *LWS* and upregulation of *RH1* expression level were also found during development in barfin flounder [33]. Similarly, no rod photoreceptors were found in retinas of larval winter flounder (*Pseudopleuronectes americanus*), indicating no expression of *RH1*, whereas three types of photoreceptors with different λ_max_, including rods, were present in adult retinas. These results indicate increased *RH1* expression and a shift in spectral sensitivity [47]. In our study of turbot, we found low expression of cone opsins and a slight increase in proportional level of *LWS* at 18 months of age. At that stage, turbot completed metamorphosis and moved to deeper waters. Thus, color vision seems not to be important for turbot in the deep sea, and higher *LWS* expression may occur in preparation for reproduction.

### Genetically based versus environmentally triggered variation

The developmental progression in opsin expression is strongly linked to shifts in spectral sensitivity, which is generally considered to be an adaptation strategy to ambient light changes that occur over the life cycle [14, 15]. Studies on bluefin killifish (*Lucania goodei*) suggest lighting environment had large effects on opsin expression [20]. However, it is reported that opsin expression plasticity in cod larvae is controlled by developmental programme rather than ambient light [5]. It is not yet clear whether heterochronic shifts in opsin expression are genetically determined or environmentally triggered. Generally speaking, opsin expression should not need to change if there are no differences in the environmental spectrum. However, in the present study, we found that the turbot, which has been artificially domesticated for a long time, still showed variation of opsin expression in the same spectral environment during different developmental stages. Earlier studies suggest that photoperiod manipulation has an impact on performance, maturation and flesh quality in turbot [48, 49]. And our lab had showed the plasticity of vision and body development of turbot larvae under different light spectra [29]. It is a pity that we have no turbot in natural environments to be compared with, whether the variation of opsin expression is triggered by environmental factors needs further study.

### The eye migration and evolutionary origin of flatfish asymmetry

Darwin had noticed the eye asymmetry of flatfish, but the evolutionary origin was unclear, because there are no transitional forms linking flatfishes to symmetrical relatives. Thus, Darwin proposed a conjecture that invoked the inheritance of acquired traits like Lamarck’s theory of flatfish origins, to respond to early arguments against natural selection. Until the discovery of the extinct spiny-finned fishes *Amphistium* and *Heteronectes*, which retain many primitive characters unknown in extant species. It indicates that the evolution of the eye asymmetry of flatfishes was gradual [50, 51]. At present, it is generally accepted that flatfishes (Pleuronectiformes) are monophyletic groups [52, 53]. If it is true, important changes for adaptation to benthic lifestyle should be shared by Pleuronectiformes. Meanwhile, a fast adaptive radiation occurring ~ 40 Mya may play a great role in evolutionary history of each family or species [30]. Our results of divergence time of turbot *RH2* genes help provide reference points.

## Conclusions

Our results indicate that evolutionary changes in gene sequences and heterochronic shifts in opsin expression are the main ways that turbot adapt to environmental photic variations from the pelagic to the benthic period. Specifically, the positive selection of E122Q and M207L of *RH2C* is closely related to a blue shift of the spectral peak absorbance, and *RH2C* may be an important green opsin gene retained by some flatfish species, including turbot, after gene duplication. In addition, spectral sensitivities tuned by heterochronic shifts in opsin expression is another strategy to adapt to different ambient light spectra during the life cycle.

## Methods

### Phylogenetic and synteny analysis

To identify the visual opsin gene repertoire of turbot, zebrafish opsin sequences were used as BLASTp query sequences with e-value < 10^−10^, and they were downloaded from the chromosome of the reference genome [30]. Opsin sequences of four flatfish (spotted halibut, *Verasper variegatus*; Japanese flounder, *Paralichthys olivaceus*; barfin flounder, *Verasper moseri*, and Atlantic halibut, *Hippoglossus hippoglossus*) and four freshwater species living in shallow water (zebrafish, *Danio rerio*; medaka, *Oryzias latipes*; guppy; and zebra mbuna, *Maylandia zebra*) were obtained from GenBank (Additional file 2) [10, 11, 33, 54, 55]. Phylogenetic relationships among the visual opsin nucleotide sequences were inferred using MEGA 7 software [56] by applying the maximum-likelihood method [57] and Kimura two-parameter model algorithm [58]. The reliability of tree topology was evaluated by bootstrap analysis with 1000 replications and uniform rates among sites. Genomicus synteny [59] and Ensembl genome browsers were used to assess the syntenic regions between turbot and five other teleost genomes (tongue sole, *Cynoglossus semilaevis*; Japanese medaka HNI; zebra mbuna; zebrafish, and guppy).

### Branch and branch-site test of selection

To estimate the differences in selection pressure between five benthic species and four shallow water pelagic species, branch-specific models and branch-site models of maximum likelihood were implemented in the CODEML program of PAML4.9i [60]. Tree topologies of each gene obtained by neighbor-joining method were provided in Additional file 1: Fig. S1. First, we compared the one-ratio model and free-ratios model in the branch test of selection [61], to test whether positive selection is present. It attempted to investigate if different spectral environments have an impact on the evolution of opsin genes. We then compared null Model A against the alternative Model A in the branch-site test to determine whether there are positively selected sites was present. This step was tried to explore the differences in selection pressure acted on not only branches but also sites of amino acids. Different sets of foreground lineages (the lineages selected for branch-site test) were marked with letters in each gene tree (Additional file 1: Fig. S1) [62]. The likelihood ratio test (LRT) was used to compare all alternative models and their corresponding null models. The Bayes Empirical Bayes method was used to obtain the posterior probability of sites under positive selection [63].

### Analysis of spectral tuning sites

Representative spectral tuning sites of amino acids were compared among the opsins of five benthic and four shallow water pelagic species. The sites obtained by site-directed mutagenesis were referenced from a previous study [10, 38]. Amino acid sequence alignments were performed using ClustalX [64]. The number of amino acid sites was standardized to bovine rhodopsin, except for blue opsin, which was standardized to barfin flounder SWS2A.

### Estimation of turbot *RH2* divergence times

The divergence time of turbot RH2 genes was estimated by MCMCTREE within PAML4.9i [60, 65]. The neighbor-joining tree of six species (turbot; Atlantic salmon, *Salmo salar*; fugu, *Takifugu rubripes*; common carp, *Cyprinus carpio*; lungfish, *Neoceratodus forsteri*, and rainbow trout) was acquired by MEGA 7 by neighbor-joining method. The fossil calibrations were adopted from TimeTree [66]. The tree topology and fossil calibrations were set as (((((((S.maximus_RH2B1, S.maximus_RH2B2), S.maximus_RH2C), T.rubripes_RH2), (S.maximus_RH2A1, S.maximus_RH2A2)), (S.salar_RH2, O.mykiss_RH2)) ‘> 1.86 < 2.27’, (C.carpio_RH2-1, C.carpio_RH2-2)) ‘> 2.05 < 2.55’, N.forsteri_RH2). The RootAge was set as ‘< 6.0’.

### Quantification of turbot opsin RNA expression

Laval and juvenile turbot were bred from a captive population at Shenghang Aquatic Science and Technology Company (Weihai, Shandong Province, China). The light period was 14L:10D and all individuals for mRNA expression analysis were euthanized with 300 mg/L of MS-222 (Sigma, Shanghai, China) between 9 and 12 am before being decapitated. The eyes were removed and immediately stored in liquid nitrogen until analyzed. Developmental stage was determined on the basis of the position of the rotating eye external characters [28]. Specifically, the ages of the individuals used for analysis of heterochronic shifts in opsin gene expression were: 0.5 month (15 days post hatch, larval stage), 1 month (metamorphic stage, the right eye has risen to the top of the head), 4, 9 and 18 months (post-metamorphic stage, asymmetric, the body color has changed to silvery gray).

Total RNA was extracted using the RNA Isolation Kit (Vazyme Biotech Co, Nanjing, China). RNA purity and concentration were examined using a NanoDrop 2000 spectrophotometer (Thermo Scientific, Shanghai, China), and RNA integrity was verified by gel electrophoresis. The PrimeScript RT reagent kit with gDNA Eraser was used to synthesize first-strand cDNA from 0.5 μg of total RNA (TaKaRa, Dalian, China). Using the Bio-Rad CFX Connect™ Real-Time PCR System (Bio-Rad, Hercules, CA, USA), quantitative real-time PCR (qPCR) was conducted with TB Green Premix Ex Taq (TaKaRa) following the manufacturer’s protocol. Melting curves were plotted to confirm amplification specificity. Using the 2^−ΔΔCt^ method, the relative expression level of each opsin gene was normalized to β-actin, which was selected as the internal reference gene after evaluating the expression pattern of eight commonly used housekeeping genes [67]. Additional file 1: Table S1 shows the specific primers for qPCR. The PCR mixture (20 µL) contained 10 μL of TB Green Premix Ex Taq, 7.6 μL of PCR-grade water, 1.6 μL of cDNA, and 0.4 μL of each of the primers. The qPCR reaction was performed in triplicate with the program of 95 °C for 3 min, followed by 40 cycles at 95 °C for 10 s, 57/58 °C for 30 s, and 72 °C for 30 s. Proportional opsin expression was determined as a fraction by calculating the proportion of each cone opsin (*T*_*i*_) relative to the total cone opsin expression (*T*_*all*_) as follows:

$$\frac{{T_{i} }}{{T_{all} }} = \frac{{\frac{1}{{\left( {1 + E_{1} } \right)^{{C_{ti} }} }}}}{{\sum {\frac{1}{{\left( {1 + E_{i} } \right)^{{C_{ti} }} }}} }}$$

where *E*_*i*_ represents the PCR efficiency for each pair of primers and *C*_*ti*_ is the critical cycle number for each gene [68, 69].

To assess significance of the change in opsin expression between different stages, the least significant difference (LSD) post hoc test with 95% confidence level was used. Data were analyzed using SPSS 23.0 software. Relative expression data are shown as the mean ± standard deviation, and proportional expression data are shown as averages.

## Supplementary Information

**Additional file 1.** Additional Figs. S1–7, Table S1–4.

**Additional file 2.** NCBI accession numbers used in this study.

## Data Availability

All NCBI accession numbers used in this study are listed in Additional file 2. The datasets used and analysed during the current study are available at Dryad: https://doi.org/10.5061/dryad.djh9w0w0m.

## Abbreviations

*RH1*: Rhodopsin
*RH2*: Rhodopsin-like opsin
*SWS1*: Short wavelength-sensitive opsin 1
*SWS2*: SWS1-like opsin
*LWS*: Long wavelength-sensitive opsin
λ_max_: Spectral peak absorbances
LRT: The likelihood ratio test
ω values: Nonsynonymous/synonymous rate ratio
N: Asparagine
D: Aspartic acid
E: Glutamic acid
Q: Glutamine
M: Methionine
L: Leucine
F: Phenylalanine
A: Alanine
S: Serine
V: Valine
T: Threonine
Mya: Million years ago
qPCR: Quantitative real-time PCR
LSD: Least significant difference
