# Supplementary material for: Evolutionary ecology of the visual opsin gene sequence and its expression in turbot (Scophthalmus maximus)
Source: BMC Ecol Evol. 2021 Jun 7;21:114. doi: 10.1186/s12862-021-01837-2 (PMC8186084; doi:10.1186/s12862-021-01837-2)
Supplement: Supplementary file 1 — Additional file 1. Additional Figs. S1–7, Table S1–4. [file 12862_2021_1837_MOESM1_ESM.doc]

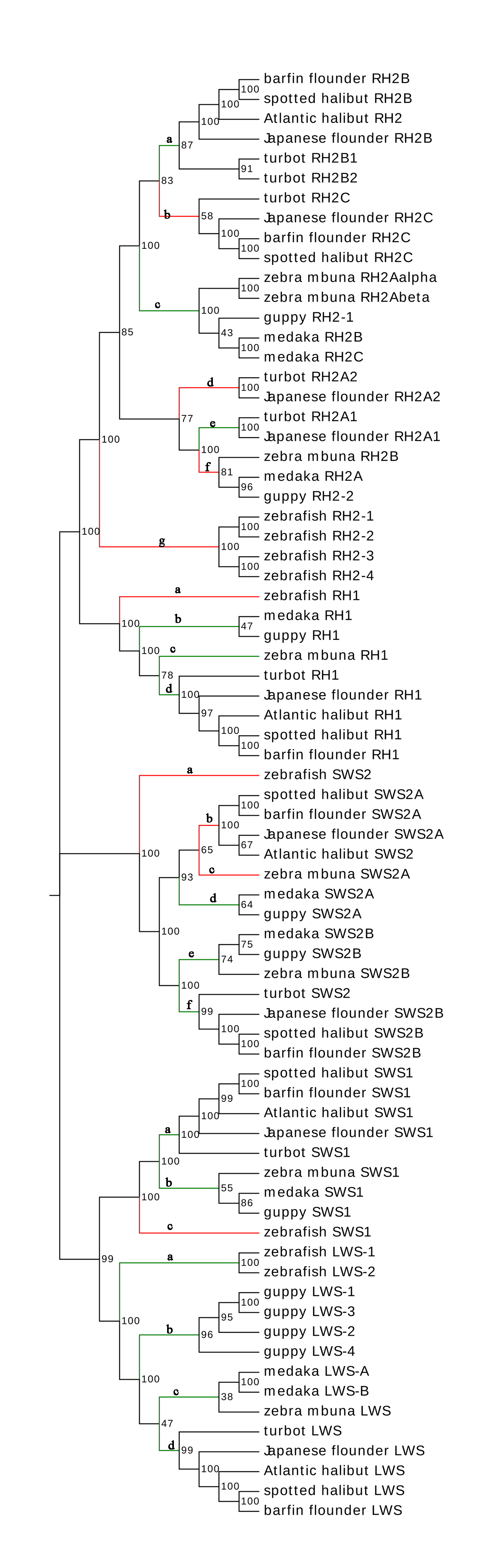


Fig. S1: Phylogenetic tree used for branch and branch-site test of selection which obtained by the neighbor-joining method. The bootstrap test (1000 replicates) scores are shown on the nodes. Branches marked in red or green were selected for branch-site models analysis: red indicates that several sites under positive selection were detected, and green indicates the opposite. Different letters marked near the branches represent the settings of the different foreground branches introduced in section 2.2.

Fig. S2: The amino acid alignments of teleost LWS opsins.

Fig. S3: The amino acid alignments of teleost RH1 opsins.

Fig. S4: The amino acid alignments of teleost RH2 opsins.

Fig. S5: The amino acid alignments of teleost SWS1 opsins.

Fig. S6: The amino acid alignments of teleost SWS2 opsins.


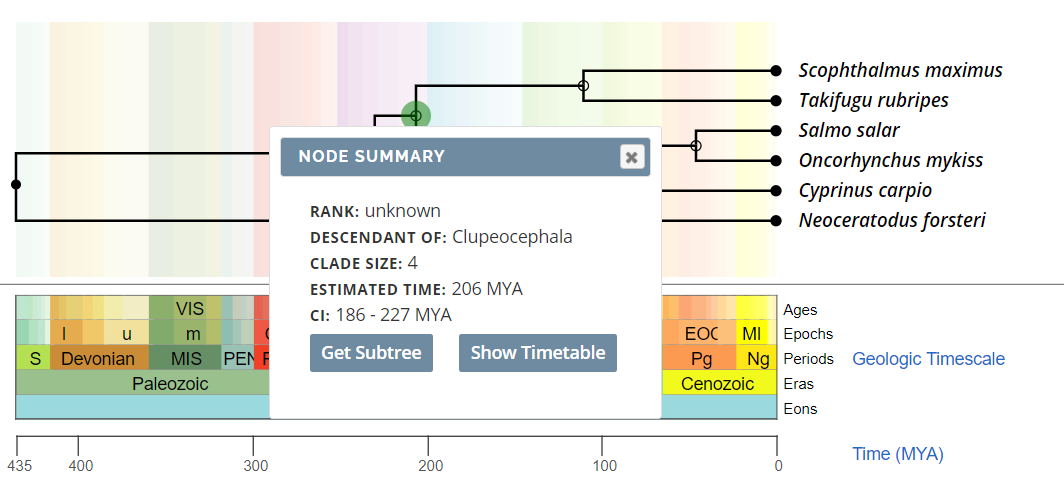

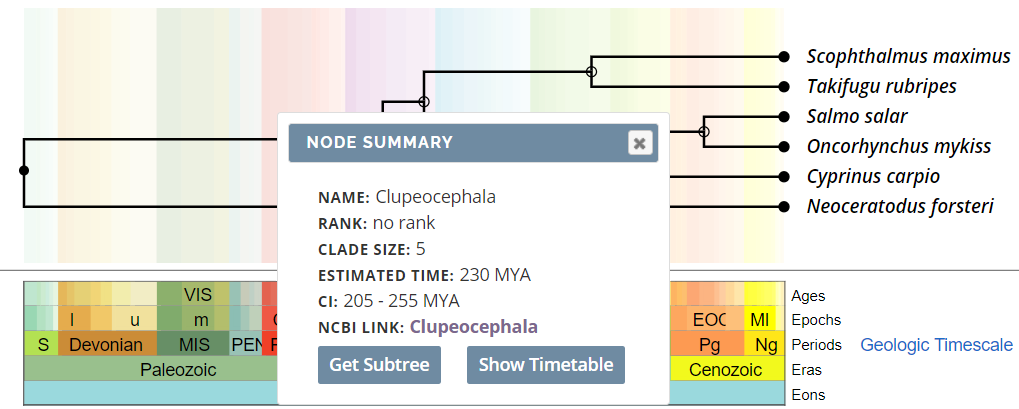


Fig. S7: The fossil calibrations adopted from TimeTree.

Table S1. Nucleotide sequences of oligonucleotide primers.

| Gene | Primer sequence (5’—3’) | PCR efficiency (%) | Annealing temperature (℃) |
| --- | --- | --- | --- |
| *RH1* | AACGACTGAAGGCTAATGT | 102.6 | 57 |
|  | AACTCTGTAATGGGCTGAC |
| *LWS* | TATTGCGTATGCTGGGGAC | 95.9 | 58 |
|  | TGGGTTGTATATGGTGGCG |
| *SWS1* | AAACACTTCCACCTGTATGAG | 100.4 | 58 |
|  | TGACGAGGATGTAGTTGAGA |
| *SWS2* | CATACCCTCCTGCCTCTCCA | 93.8 | 58 |
|  | AATCCTCATCATCGCCTCCA |
| *RH2A1* | GCAGTCAAACGATTCCCAT | 98.0 | 58 |
|  | GTTCCTCTAACAACCACCAAAA |
| *RH2A2* | CTATCCACCAGCAAGACAGAAG | 105.3 | 58 |
|  | CCATTTCAAGTTAGCCATTCAG |
| *RH2B1* | TTGACCAGTGACGAATGGCTCC | 95.1 | 57 |
|  | GCTGAGAGATTCCTGTTCCCCG |
| *RH2B2* | TTACCGATTGCTTCCAGATTTC | 99.3 | 58 |
|  | ATGAGGCGGCATTCCTTTGTGT |
| *RH2C* | CTGTGGACGCTCTCCTTGAC | 99.9 | 57 |
|  | GACTTGCGAGTTGAGGTGAA |

Table S2. Comparison of representative spectral tuning sites among teleost SWS2 opsins.

| Tuning site | | 52 | 55 | 58 | 99 | 100 | 122 | 124 | 170 | 213 | 275 | 301 |
| --- | --- | --- | --- | --- | --- | --- | --- | --- | --- | --- | --- | --- |
| spotted halibut | SWS2A | F | V | T | V | G | M | T | A | L | T | S |
|  | SWS2B | F | V | T | T | C | T | T | A | L | A | S |
| barfin flounder | SWS2A | F | V | T | V | G | M | T | A | L | T | S |
|  | SWS2B | F | V | T | T | C | T | T | A | L | A | S |
| Atlantic Halibut | RH2 | F | V | T | V | G | M | T | A | L | A | S |
| Japanese flounder | SWS2A | F | V | T | V | G | M | T | A | L | A | S |
|  | SWS2B | F | V | T | T | C | T | T | A | L | A | S |
| turbot | SWS2 | F | V | T | T | C | S | T | A | L | A | S |
| cichlids | SWS2A | F | I | T | T | A | M | T | A | L | A | S |
|  | SWS2B | F | V | T | T | C | T | S | A | L | A | S |
| medaka | SWS2A | F | V | T | T | A | A | T | A | L | A | S |
|  | SWS2B | V | V | T | T | C | T | A | A | L | A | S |
| guppy | SWS2A | F | V | T | T | T | V | T | A | L | A | S |
|  | SWS2B | F | V | T | T | C | T | T | A | L | A | S |
| zebrafish | SWS2 | F | I | T | V | A | T | T | A | L | A | C |

Table S3. Comparison of representative spectral tuning sites among teleost SWS1 opsins.

| Tuning site | 46 | 49 | 52 | 86 | 90 | 93 | 114 | 118 |
| --- | --- | --- | --- | --- | --- | --- | --- | --- |
| spotted halibut | S | F | T | F | S | Q | S | S |
| Barfin flounder | S | F | T | F | S | Q | S | S |
| Atlantic Halibut | S | F | T | F | S | Q | S | S |
| Japanese flounder | S | F | T | F | S | Q | S | S |
| turbot | F | F | T | F | S | Q | A | S |
| medaka | F | F | T | F | S | Q | A | A |
| guppy | F | F | T | F | S | Q | A | A |
| cichlids | F | F | T | F | S | Q | S | S |
| zebrafish | F | I | T | F | S | Q | A | S |

Table S4. Comparison of representative spectral tuning sites among teleost LWS opsins.

| Tuning site | | 164 | 181 | 261 | 269 | 292 |
| --- | --- | --- | --- | --- | --- | --- |
| spotted halibut | LWS | A | H | Y | T | A |
| Barfin flounder | LWS | A | H | Y | T | A |
| Atlantic Halibut | LWS | S | H | Y | T | A |
| Japanese flounder | LWS | A | H | Y | T | A |
| turbot | LWS | P | H | Y | T | A |
| cichlids | LWS | A | H | Y | T | A |
| medaka | LWS-A | S | H | Y | T | A |
|  | LWS-B | S | H | Y | T | A |
| guppy | LWS-1 | A | H | Y | T | A |
|  | LWS-2 | P | H | F | A | A |
|  | LWS-3 | S | H | Y | T | A |
|  | LWS-4 | S | H | Y | T | A |
| zebrafish | LWS-1 | A | H | Y | T | A |
|  | LWS-2 | A | H | F | T | A |
