# Supplementary material for: Evolutionary ecology of the visual opsin gene sequence and its expression in turbot (Scophthalmus maximus)
Source: BMC Ecol Evol. 2021 Jun 7;21:114. doi: 10.1186/s12862-021-01837-2 (PMC8186084; doi:10.1186/s12862-021-01837-2)
Supplement: Supplementary file 2 — Additional file 2. NCBI accession numbers used in this study. [file 12862_2021_1837_MOESM2_ESM.docx]

NCBI accession numbers used in this study.

*Verasper variegatus* (LWS: LC209595; RH2-B: LC209601; RH2-C: LC209604; SWS2A: LC209609; SWS2B: LC209612; SWS1: LC209598; RH1: LC209606)

*Verasper moseri* (LWS: AB930175; RH2B: AB930177; RH2C: AB930178; SWS2A: AB930180; SWS2B: AB930181; SWS1: AB930179; RH1: AB930176)

*Paralichthys olivaceus* (LWS: LC209597, RH2-A1; LC209812; RH2-A2: LC209813; RH2-B: LC209603; RH2-C: LC209605; SWS2A: LC209611; SWS2B: LC209614; SWS1: LC209600; RH1: LC209608)

*Hippoglossus hippoglossus* (RH2: AF156263; RH1: AF156265; LWS: AF316498; SWS1: AF156264; SWS2: AF316497).

*Scophthalmus maximus* (LWS: AF385826.1; RH1: MN073188; SWS1: AWO98359.1; SWS2: AWP03347.1; RH2A1: MN073190; RH2A2: MN073191; RH2B1: MN073192; RH2B2: MN073193; RH2C: MN073194)

*Danio rerio* (RH2: NM_131253, NM_182891, NM_182892, and NM_131254; SWS2: NM_131192; LWS: NM_001313715, NM_001002443; SWS1, BC060894; RH1, NM_131084)

*Oryzias latipe* (RH1: AB180742; LWS: AB223051 and AB223052; RH2: AB223053, AB223054, and AB223055; SWS2: AB223056 and AB223057; and SWS1: AB223058)

*Maylandia zebra* (SWS2 and LWS: JF262084, SWS1: JF262085, RH2: JF262089, RH1: AY775114)

*Poecilia reticulata* (RH1: LC127191; LWS: AB748984, LC127183, LC127184, LC127185; RH2: DQ234858 and DQ234859; SWS2: JF303638 and DQ234860,SWS1: LC127190,)

*Takifugu rubripes* (RH2: NM_001033712), *Salmo salar* (RH2: NM_001123707), *Oncorhynchus mykiss* (RH2: NM_001124323), *Cyprinus carpio* (RH2: AB110602 and AB110603), *Neoceratodus forsteri* ( RH2: EF526296).

**References**

1. Kasagi S, Mizusawa K, Takahashi A. Green-shifting of SWS2A opsin sensitivity and loss of function of RH2-A opsin in flounders, genus *Verasper*. Ecol Evol. 2018;8:1399-410.
2. Kasagi S, Mizusawa K, Takahashi A. Green-shifting of SWS2A opsin sensitivity and loss of function of RH2-A opsin in flounders, genus *Verasper*. Ecol Evol. 2018;8:1399-410.
3. Helvik JV, Drivenes O, Naess TH, Fjose A, Seo HC. Molecular cloning and characterization of five opsin genes from the marine flatfish Atlantic halibut (Hippoglossus hippoglossus). Visual Neuroscience. 2001;18:767-80.
4. Laver CRJ, Taylor JS. RT-qPCR reveals opsin gene upregulation associated with age and sex in guppies (Poecilia reticulata): a species with color-based sexual selection and 11 visual-opsin genes. BMC Evol Biol. 2011;11:81.
5. Nakamura Y, Mori K, Saitoh K, Oshima K, Mekuchi M, Sugaya T, et al. Evolutionary changes of multiple visual pigment genes in the complete genome of Pacific bluefin tuna. PNAS. 2013;110:11061-6.
